# Rapid Encoding and Perception of Novel Odors in the Rat

**DOI:** 10.1371/journal.pbio.0060082

**Published:** 2008-04-08

**Authors:** Daniel W Wesson, Ryan M Carey, Justus V Verhagen, Matt Wachowiak

**Affiliations:** 1 Department of Biology, Boston University, Boston, Massachusetts, United States of America; 2 Department of Biomedical Engineering, Boston University, Boston, Massachusetts, United States of America; Boston University, United States of America

## Abstract

To gain insight into which parameters of neural activity are important in shaping the perception of odors, we combined a behavioral measure of odor perception with optical imaging of odor representations at the level of receptor neuron input to the rat olfactory bulb. Instead of the typical test of an animal's ability to discriminate two familiar odorants by exhibiting an operant response, we used a spontaneously expressed response to a novel odorant—exploratory sniffing—as a measure of odor perception. This assay allowed us to measure the speed with which rats perform spontaneous odor discriminations. With this paradigm, rats discriminated and began responding to a novel odorant in as little as 140 ms. This time is comparable to that measured in earlier studies using operant behavioral readouts after extensive training. In a subset of these trials, we simultaneously imaged receptor neuron input to the dorsal olfactory bulb with near-millisecond temporal resolution as the animal sampled and then responded to the novel odorant. The imaging data revealed that the bulk of the discrimination time can be attributed to the peripheral events underlying odorant detection: receptor input arrives at the olfactory bulb 100–150 ms after inhalation begins, leaving only 50–100 ms for central processing and response initiation. In most trials, odor discrimination had occurred even before the initial barrage of receptor neuron firing had ceased and before spatial maps of activity across glomeruli had fully developed. These results suggest a coding strategy in which the earliest-activated glomeruli play a major role in the initial perception of odor quality, and place constraints on coding and processing schemes based on simple changes in spike rate.

## Author Summary

Olfactory stimuli elicit temporally complex patterns of activity across groups of receptor neurons as well as across central neurons. It remains unclear which parameters among these complex activity patterns are important in shaping odor perception. To address this issue, we imaged from the olfactory bulb of awake rats as they detected and responded to odorants. We used a spontaneously expressed response to novel odorants—exploratory sniffing—as a behavioral measure of odor perception. This assay allowed us to measure the speed with which rats perform simple odor discriminations by monitoring changes in respiration. Rats discriminated a novel odorant from a learned one in as little as 140 ms. Simultaneously imaging the sensory input to the olfactory bulb carried by receptor neurons revealed that the bulk of the response time is due to the peripheral events underlying odorant detection (inhalation and receptor neuron activation), leaving only 50–100 ms for central processing and response initiation. In most trials, responses to a novel odorant began before the initial barrage of input had ceased and before spatial patterns of input to the bulb had fully developed. These results suggest a coding strategy in which the earliest inputs play a major role in the initial perception of odor quality and place constraints on coding schemes based on simple changes in firing rate.

## Abstract 3

Imaging the olfactory bulb of awake rats reveals that odor discrimination occurs about 100 ms after sensory input reaches the brain, sharply limiting the role that spike rate and temporal integration can play in coding odor identity.

## Introduction

Information about olfactory stimuli (odorants) is reliably represented by multiple parameters of neural activity. Different odorants evoke unique patterns of spiking across populations of olfactory receptor neurons (ORNs), which in turn lead to unique spatial patterns of activated glomeruli in the olfactory bulb (OB) [1–3]. These patterns are easily visualized as spatial maps of activity in the OB and are thought to play a primary role in coding odor information [2,3]. Different odorants also evoke unique temporal patterns of neural activity in ORNs, OB neurons, and olfactory cortex neurons; these temporal patterns have also long been hypothesized to play a role in odor coding [4,5]. Finally, spatial maps of glomerular activity are themselves temporally dynamic, changing in an odorant-specific manner over the course of tens to hundreds of milliseconds [5–8]; much of these dynamics are organized relative to the respiratory cycle, which controls the bulk flow of odorant into the nasal cavity with each inhalation [9,10]. These temporal dynamics of glomerular activation have also been hypothesized to play a role in odor coding [5]. The role that spatial and temporal parameters of odorant-evoked activity play in shaping odor perception remains unclear.

Behavioral assays are an important tool for approaching this problem because they can define perceptual limits and constrain which features of neural activity might underlie perception. For example, recent studies investigating the speed with which animals perform simple odor discriminations have found that mice and rats can perform such tasks with high accuracy in 200–300 ms [11–14]. These results have been interpreted as constraining the role of temporal dynamics in odor coding, as many (though not all) time-dependent coding schemes require more than a few hundred milliseconds to operate optimally [4,5,8,15]. Interpreting these studies has several limitations, however. First, response times depend on many factors unrelated to the sensory task itself, such as the choice of behavioral paradigm used, the specific behavioral response required, stimulus valence, or even foreperiod duration [14,16,17]. Second, reported odor discrimination times are based on discrimination of a single pair of odorants after an extensive training period; the simple nature of this task could lead to response-time measurements unrepresentative of the animal's natural behavior, and the training phase of this paradigm has the potential to alter the neural strategies underlying the discriminative process itself.

It would thus be informative to measure response times for a spontaneous (i.e., unlearned) test of odor perception. In addition, behavioral assays have traditionally yielded the most insight into neural coding strategies when actually paired with simultaneous measurements of neural activity [18–21]; such an analysis has not yet been performed in the context of odor response times. Here, we analyze the timing of odor perception using a spontaneously expressed and ethologically natural odor discrimination behavior: the expression of high-frequency exploratory sniffing in response to a novel odorant. In a subset of behavioral trials, we simultaneously imaged ORN input to the OB with near-millisecond temporal resolution using calcium-sensitive dyes. Imaging allowed us to make separate estimates of the time needed for transmission of sensory signals to the brain and the time needed for the central events underlying odor discrimination and response initiation. Surprisingly, we find that the spontaneous (i.e., unreinforced) discrimination of a novel odor from a learned one can occur in as little as 150 ms and in most cases occurs in less than 200 ms. Thus, rodents can perform spontaneous odor discriminations at least as rapidly as they perform highly trained ones [11–13]. We also find that the behavioral response begins as soon as 50 ms after the arrival of sensory input to the brain, indicating that the central processing underlying this discrimination is extremely rapid and is complete even before spatial maps of glomerular activity have fully developed. These results place new constraints on the neural mechanisms underlying elementary odor discriminations, and suggest that alternatives to simple rate-based coding and processing schemes are needed to explain such rapid discriminations.

## Results

We trained 13 rats to perform a simple lick/no-lick two-odor discrimination task using a head-fixed behavioral paradigm. The head-fixed paradigm allowed us to image receptor input to the dorsal OB during the discrimination task, and to monitor respiration via a chronically implanted intranasal cannula [22]. Restricting movement also allowed us to present olfactory stimuli to the animal with high fidelity and temporal precision, and eliminated movement-driven effects on respiratory behavior (such as approach to a sampling port) which can confound interpretation of respiratory responses to sensory stimuli [23]. We have characterized respiratory behavior and odor discrimination performance in head-fixed rats in a previous report [22]; briefly, rats performed the task with high (>85%) accuracy and showed baseline respiratory behavior typical of unrestrained, inactive rats in a familiar environment [24], with a mean respiration rate of 1–2 Hz.

### Spontaneous Discrimination of Novel and Familiar Odorants

Rats showed little change in respiration rate when discriminating two learned odorants [22], but showed robust changes when presented with a novel (i.e., not previously presented) odorant (Figure 1A). The typical response to a novel odorant consisted of a bout of high-frequency respiration showing peak frequencies of 6–8 Hz (Figure 1B) and typically lasting for several seconds (3.92 ± 2.11 s, mean ± standard deviation [s.d.]). This behavior is well described in unrestrained rodents, and has been termed “exploratory sniffing” [25–28]. Exploratory sniffing in the head-fixed rat was qualitatively distinct from other modes of high-frequency sniffing, with a longer duration and lower peak frequency than sniffing behavior associated with reward anticipation or approach to an odorant port [23,29].

**Figure 1:**
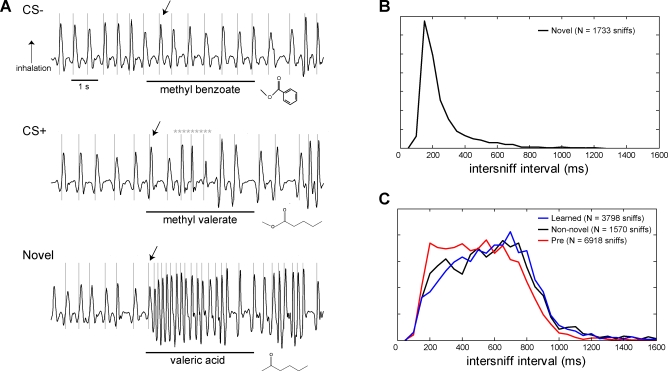
Exploratory Sniffing Evoked by Novel Odorants (A) Respiratory behavior in a head-fixed rat performing a lick/no-lick discrimination of two familiar odorants (top two traces) and during presentation of a novel odorant (bottom trace). Inhalation is up. Vertical lines mark the start of each inhalation. Arrows mark the first inhalation in the presence of odorant. Asterisks in middle trace show time of licking to receive water reward. There is no appreciable change in respiration in response to the learned odorants, but the novel odorant elicits high-frequency, exploratory sniffing. (B) Distribution of ISIs for all inhalations during novel odorant presentations. (C) Distribution of ISIs for all presentations of learned odorants (blue), all third and subsequent presentations of novel odorants (black), and all pre-odor epochs (red). Plots are based on histogram distributions with 50-ms time bins.

To classify sniffing behavior on a trial-by-trial basis, we defined exploratory sniffing as a bout of sniffing consisting of at least nine sniffs within any 2-s block of the odorant presentation (thus, a minimum average frequency of 4 Hz). Using this criterion, rats showed exploratory sniffing to 89% (67/75) of all first presentations of a novel odorant, compared with only 3% (14/472) of all presentations of learned (rewarded [CS+] and unrewarded [CS−]) odorants and 5% (36/768) of all pre-odor epochs. Likewise, mean respiration rate (measured as intersniff interval [ISI]) for all novel presentations was significantly higher (246 ± 86 ms, *n* = 74 trials) than for either learned-odorant trials (612 ± 160 ms, *n* = 472 trials, *p* = 9 × 10^−64^, Student's *t*-test) or pre-odor epochs (556 ± 215 ms, *n* = 767 trials, *p* = 3 × 10^−34^) (Figure 1C). As previously reported [22], rats rapidly habituated to subsequent presentations of the novel odorant, with respiratory behavior becoming indistinguishable from that of learned-odorant trials by the third presentation (Figure 1C). Thus, the expression of exploratory sniffing during the time of odorant presentation was a reliable behavioral indicator that the rat had discriminated a novel odorant from a learned one.

### Latency to Exploratory Sniffing Reveals Rapid Odor Perception

Another striking feature of the exploratory sniffing response was the speed with which it was initiated. In the example of Figure 1A, the exploratory sniff bout begins approximately 220 ms after the first inhalation of odorant. To further characterize response times to novel odorants, we compared respiratory behavior in all novel trials with that in all trials involving the presentation of a learned (CS−) odorant. CS− trials with no licking were used for this comparison to eliminate the possibility that licking during CS+ trials affected respiration (an example of such an effect is apparent in Figure 1A). Trials showing exploratory sniffing before odorant onset (3/75 novel trials, 11/187 CS− trials) were also excluded. All other novel trials were included, regardless of whether respiratory behavior met the criteria for exploratory sniffing. Data were pooled across animals and sessions because of the small number of novel-odorant presentations per session (*n* = 1–4).

We determined the time to discriminate a novel from a learned odorant from the time course with which the cumulative sniff count became reliably different for the two odorants (Figure 2A). We used an analytical approach similar to that used to measure response times in a nose-poke–based, go/no-go odor discrimination paradigm [11] and in previous analyses of odorant-evoked changes in human respiratory behavior [30]. Time zero was set to the time of the first inhalation after odorant onset rather than the time of odorant onset, due to the low (1–2 Hz) resting respiration rate exhibited in the head-fixed rats and on the assumption that inhalation is required for odorant to be detected by the animal. This assumption was validated in the imaging experiments described below. Sniff counts for novel- versus learned-odorant trials diverged rapidly, becoming significantly different (*p* < 0.05) after 140 ms (Figure 2B). This value represents the *minimum* time at which reliably distinct behavioral responses to novel and learned odorants—averaged across many trials—emerge. Thus, distinct behavioral responses to novel and learned odorants can emerge in well under 200 ms. Repeating this analysis with time zero set to the time of odorant onset led to divergent respiratory behavior at 295 ms. In contrast, discrimination times measured using the operant lick response for CS+ trials were longer and more variable, with a median time of 730 ms (±890 ms, s.d.; *n* = 184) from the first inhalation after odorant onset to the start of licking.

**Figure 2:**
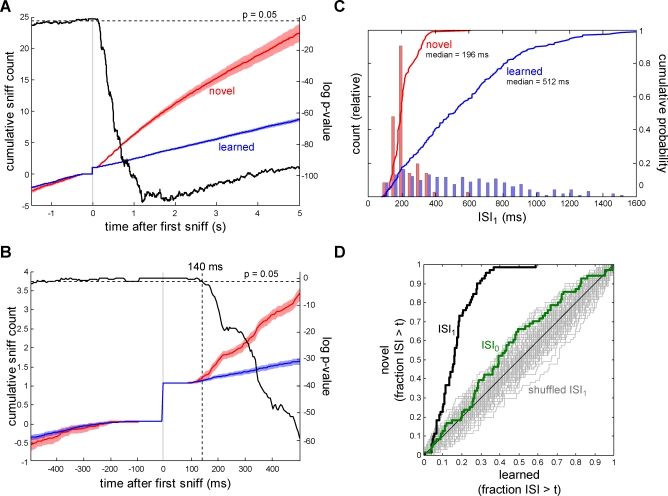
Respiration Changes Rapidly after Detection of a Novel Odorant (A) Cumulative sniff counts (left axis) for novel (red) and learned (blue) odorant presentations. Time bin is 5 ms. Solid line indicates the mean value, shaded areas indicate standard error of the mean (s.e.m.). Sniff count for each trial is set to one at the time of the first inhalation after odorant onset (time = 0). Inhalations preceding odorant onset (negative time) are counted as negative in the cumulative sniff count, to track respiration preceding odorant presentation. Black plot shows log *p*-value (right axis) of Student's *t*-test comparison of novel and learned trials. (B) Detailed view of same plots as in (A), around time of odorant detection. Vertical dashed line indicates time after which the *p*-value remains below 0.05 (140 ms). (C) Comparison of ISI between the first inhalation after odorant onset (i.e., the “detection sniff”) and the next inhalation (ISI_1_) for novel and learned trials. The two distributions differ dramatically. Time bin is 50 ms. (D) ROC curves comparing the relative distributions of ISI values between novel and learned trials, for both ISI_1_ (black) and for the first inhalation and the one preceding it (ISI_0_, green). Diagonal line represents identical distributions of novel and learned values. Gray traces indicate ROC curves for all ISI_1_ data randomly designated as novel or learned (100 iterations); the ISI_1_ curve is far above the diagonal, indicating a strong separation between the distributions. The ISI_0_ curve is within the range of chance indicated by the shuffled trials.

Given that the respiration frequency at the time of odorant sampling is 1–2 Hz, this result implies that novel odorants are identified as such after only a single inhalation. Indeed, the time from the first inhalation after odorant onset (signifying the start of odorant sampling) to the second inhalation for all novel-odorant trials had a median value of 196 ms (±80 ms, s.d.; *n* = 71). This “first ISI” (ISI_1_) was significantly longer for learned odorants (median, 512 ms; *n* = 246; Kolmogorov-Smirnov test, *p* < 10^−15^; Figure 2C). Only one of the 71 novel trials had an ISI_1_ value greater than the median ISI_1_ of learned trials. As a control, we measured the ISI immediately *preceding* the first odorant inhalation (ISI_0_). This interval did not differ significantly between novel and learned trials (Kolmogorov-Smirnov test, *p* > 0.05). Thus, the spontaneous discrimination of novel versus familiar odorants typically occurs after a single inhalation.

To estimate the reliability with which the second inhalation represented the actual behavioral response to the novel odorant (as opposed to a spontaneously expressed short ISI_1_), we constructed a receiver operating characteristic (ROC) graph [31] comparing the distributions of ISI_1_ values for novel and learned trials (Figure 2D). The ROC graph plots the fraction of ISI_1_ values below a particular time *t* obtained in learned-odorant trials with the fraction of ISI_1_ values below *t* for novel trials. The cutoff time *t* is increased incrementally until all trials have been included in the graph. The slope of the ROC graph at a given point reflects the ratio of the fraction of ISI_1_ values below *t* for learned versus novel odorants [31], and thus the probability that a given ISI_1_ value occurred during learned versus novel trials. The diagonal of this graph thus indicates an equal probability that ISI values below a particular time threshold occurred on novel and non-novel trials; deviations above the diagonal indicate an increased probability that lower ISI values arose from novel trials. The ROC graph for ISI_0_ values (the ISI preceding odorant presentation) was close to the diagonal and had a slope of approximately 1 across most of its distribution. In contrast, the ROC graph for ISI_1_ values was well above the diagonal and, for the ISI_1_ values below 400 ms (corresponding to all but one novel trial), had a slope slightly greater than 3. This slope reflects an approximately 3:1 likelihood that ISI_1_ values below 400 ms occurred during novel trials than during non-novel trials. It follows that approximately one of four novel ISI_1_ values reflected spontaneous changes in respiratory behavior rather than responses to the novel odorant. By analogy to two-choice operant behavioral tasks, this relationship reflects a “false-positive” rate of 25%. Note that, in such a two-choice task, a false-positive rate of 25% would result in a measured performance accuracy of 87.5% (since 25/2 = 12.5% of all false-positive trials would result in “correct” performance)—a level near-to-above performance criterion in many earlier studies of odor discrimination ability [14,23,32]. Thus, for novel-odorant presentations, the duration of the first ISI after odorant presentation is a reliable measure of odor discrimination time.

Finally, we asked whether ISI_1_ values varied depending on the structural similarity between the novel and learned (either the CS+ or CS−) odorants. Odorants classified as similar included enantiomers and aliphatic hydrocarbons with the same functional group but differing either in carbon chain length or functional group position by one carbon (Table 1); earlier studies have provided behavioral evidence that odorants with these structural similarities are perceived as similar by rodents [33,34]. ISI_1_ values for similar and dissimilar odorants were indistinguishable (Kolmogorov-Smirnov test, *p* = 0.39; see also Table 1). Every presentation of a “similar” novel odorant elicited an ISI_1_ that was shorter than the median of all learned presentations. Similar odorants were also highly effective at eliciting exploratory sniffing according to the nine sniffs/2 s criterion (21/25 = 84% for similar odorants; 43/47 = 91% for dissimilar odorants). Thus, even odorants that are structurally and perceptually similar to each other are spontaneously and rapidly identified as novel in our behavioral paradigm.

**Table 1:**
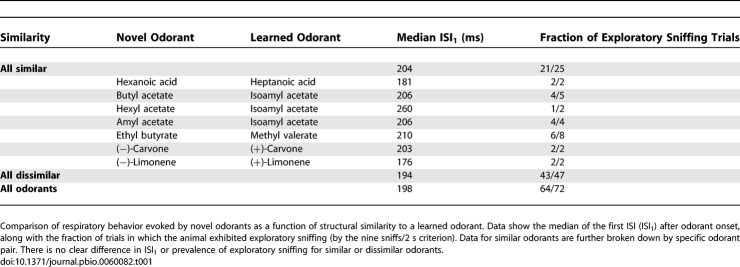
Comparison of Respiratory Behavior Evoked by Novel Odorants as a Function of Structural Similarity to a Learned Odorant.

### Estimating Olfactory Processing Dynamics Using Optical Probes

Olfactory response times include the time required for the peripheral events underlying odor detection (including bulk flow of odorant into the nasal cavity, odorant transduction, and action potential conduction from the epithelium to the OB) as well as the central events underlying odor discrimination and generation of a behavioral response. We used optical signals reflecting the arrival of action potentials at the axon terminals of the ORNs [35,36], imaged in a subset of the same trials as in our behavioral analysis, to estimate the times required for the peripheral versus central components of odor discrimination. Figure 3A and 3B shows examples of respiration and optical signals measured from two glomeruli activated by an odorant (ethyl butyrate, 1% saturated vapor [s.v.]) presented for the first time to an awake rat. As previously described [22], the optical signals indicate that ORNs are activated transiently and reliably after each sniff during low-frequency respiration (Figure 3B, late portion of traces), but show attenuation in response amplitude during exploratory sniffing (Figure 3B, early portion of traces). In most cases, odorants evoked large-amplitude signals in several glomeruli (Figure 3A). Some odorants failed to evoke strong input to any imaged glomeruli—either they evoked small-amplitude signals or none. We also observed that different glomeruli could become activated with slightly different latencies and rise times of the calcium signal (Figure 3C), as we have reported previously in anesthetized mice [6].

**Figure 3:**
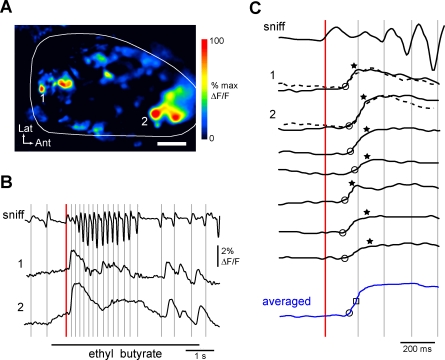
Timing of Exploratory Sniffing Relative to Receptor Input to the Olfactory Bulb (A) “Sniff-triggered average” map of presynaptic calcium signals reflecting ORN input to glomeruli of the dorsal OB evoked by ethyl butyrate (1% s.v.). Map is the average of 31 sniffs in eight trials. “1” and “2” indicate the location of glomeruli whose optical signals are shown in (B) and (C). Scale bar indicates 500 μm. Ant, anterior; Lat, lateral. ΔF, change in fluorescence; F, resting fluorescence. (B) Respiration traces (“sniff,” top) and optical signals from two glomeruli during the first presentation of ethyl butyrate. Vertical lines indicate onset times of each inhalation; red line is the first inhalation after odor onset. Responses become attenuated during high-frequency exploratory sniffing and recover as sniff frequency slows. (C) Expansion of the time around the first sniff after odor onset in (B), showing the responses of the two glomeruli from (A) and (B), plus five additional glomeruli. The latency from the first inhalation to response onset (circle) is approximately 120 ms for each glomerulus; the next inhalation marking the beginning of exploratory sniffing occurs at or before either optical response reaches 90% maximum (star). For glomeruli 1 and 2, dashed traces show the response evoked by the first inhalation of another presentation of ethyl butyrate that did not elicit exploratory sniffing, demonstrating a similar latency and rise time in the optical signal. The blue trace represents the averaged, normalized response of the seven responding glomeruli; the times of the signal onset (circle) and 50% maximum (square) are indicated.

To measure the time required for the peripheral component of odor discrimination (i.e., odorant detection), we first sought to confirm our assumption that the appropriate time to begin this measurement was the beginning of an inhalation, rather than the time at which the odorant was presented. This analysis was meant to test the possibility that odorant directed at the nose might enter the nasal cavity even in the absence of a detectable inhalation. To test for this, we examined 43 trials (including ten odorants from five animals) in which no inhalation occurred in a 250-ms window around the odorant onset (100 ms before to 150 ms after odorant onset). Visual inspection of these trials revealed no clear optical signal increases that occurred after odorant onset, but before the first inhalation. We confirmed this result by extracting the optical signals from all responding glomeruli for each of the 43 trials, and normalizing and aligning these traces either to the time of odorant onset or the time of the onset of the first inhalation (Figure 4). The top trace in Figure 4 shows the average optical signal aligned to odorant onset, along with the subsequent inhalation times for each trial; there is no statistically significant increase in the signal at any time before the first inhalation (one-tailed Student's *t*-test comparing maximum signal in the first 150 ms after odorant onset with mean signal in the 100 ms prior to odorant onset, *p* = 0.21). In contrast, aligning the traces to the inhalation time reveals a strong increase in the mean optical signal beginning approximately 120 ms after inhalation (Figure 4, bottom). We thus conclude that inhalation is required for odorant-evoked activation of ORNs.

**Figure 4:**
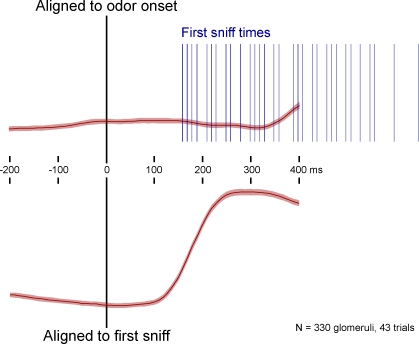
Inhalation Is Required for Odorant-Evoked Activation of Olfactory Receptor Neurons Traces show averaged, normalized optical signals from responding glomeruli in each of 43 trials in which there were no inhalations from 100 ms prior to odorant onset to at least 150 ms after odorant onset. Top trace shows average signal after aligning each trace to the time of odorant onset. The time of first inhalation for each trial is indicated by a vertical line. Bottom trace shows the same data but aligned to the time of inhalation onset. Shaded area indicates standard error. There is no increase in either signal amplitude or variance around the time of odorant onset (top), but there is a clear increase beginning approximately 100 ms after inhalation (bottom).

Odorant detection time can thus be defined as the time from the start of an inhalation to the time of arrival of action potentials at the ORN terminal in the olfactory bulb. To identify this time using imaged presynaptic calcium signals, we used a custom algorithm (see Materials and Methods) that identified the onset time of the increase in the optical signal following an inhalation (shown in Figure 3C). We confirmed that the onset times of odorant-evoked calcium signals reasonably reflected the time of arrival of action potentials at the ORN terminals by measuring onset times of calcium signals evoked by a single, brief (0.1 ms) electrical shock delivered to the olfactory nerve layer in anesthetized rats. For these experiments, optical signals were acquired at 125-Hz frame rate (8-ms frame interval) to increase temporal resolution. ON shock-evoked responses had similar amplitudes and signal-to-noise ratios as odorant-evoked signals. Onset times were measured for nine glomeruli (two preparations) ranging in distance 100–600 μm from the stimulating electrode. In all nine glomeruli, the time from ON shock to response onset was one frame (8 ms). Thus, presynaptic calcium signals measured from ORN terminals in vivo follow incoming action potential dynamics with a delay of 8 ms or less. This finding is consistent with earlier data from the turtle olfactory bulb, in which ON shock-evoked presynaptic calcium signals in ORNs lagged the compound action potential imaged with voltage-sensitive dye by approximately 2 ms [37].

Having established a reliable measure for the time required for odorant detection, we next measured this time for all imaged trials in which the first inhalation beginning at least 30 ms after odorant onset evoked a reliable response (signal-to-noise ratio greater than 4:1) in at least one glomerulus (see Materials and Methods). To determine whether the first inhalation evoked a response, we looked for optical signal onsets in the first 225 ms after inhalation began. In a previous analysis of inhalation-response latencies that used slightly different signal detection criteria, manual selection of responses, and a smaller and different dataset [22], we found response latencies to be 110 ± 28 ms (mean ± s.d.); thus, our time window represents approximately the mean ± 4 s.d. of this earlier-measured value. This criterion was met by 21 of 33 imaged novel trials (ten different odorants, 14 sessions, and seven animals) and 112 of 168 learned CS− trials (11 odorants, 21 sessions, and seven animals).

We defined odorant detection time (*t*_detect_) as the latency from the beginning of the first inhalation to the onset of the optical signal in OB glomeruli. Figure 3C shows this time period in more detail for the sample data shown in Figure 3B. We first used only the earliest response onset (i.e., the fastest-responding glomerulus) to determine a single value of *t*_detect_ for that trial. We combined measurements from novel- and learned-odorant presentations for this analysis (novel odorants cannot be discriminated prior to detection: a comparison of *t*_detect_ values by novelty showed no difference in distribution; Kolmogorov-Smirnov test, *p* = 0.20). The mean detection time was 120 ± 45 ms (mean ± s.d.; *n* = 133; Figure 5A). Surprisingly, this time is similar to the minimal response time based on the cumulative sniff count (140 ms, Figure 2B), leaving little time for central processing or motor action.

**Figure 5:**
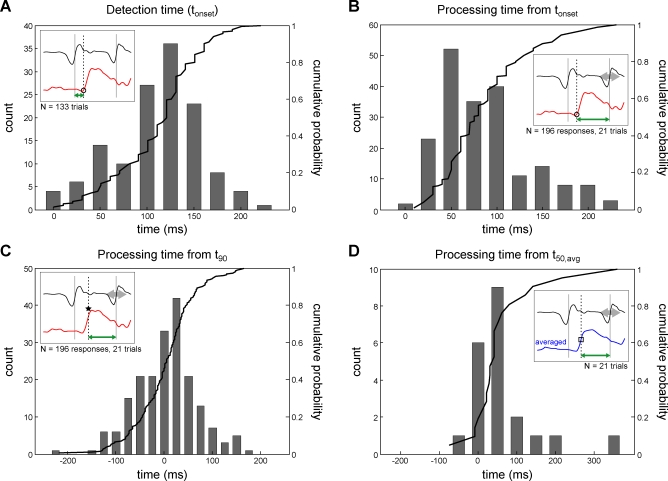
Odorant Detection and Processing Times Estimated from Optical Imaging of Receptor Inputs (A) Histogram and cumulative probability plot of optical signal-onset latencies measured from all imaged trials (median, 120 ms). Time bin is 25 ms. In all panel insets, green arrows and vertical dotted lines indicate the interval measured in the panel, overlaid on sample respiration (black) and either optical response (red) or averaged response (blue) traces. Circle indicates response onset time. (B) Histogram and cumulative probability plot of processing times based on the time from optical signal onset to the start of the second inhalation (median, 75 ms). Time bin is 25 ms. (C) Histogram and cumulative probability plot of processing times based on the time of the 90% maximum of the optical signal (star in optical signal trace). Many values are negative (median, 6 ms), indicating that the next sniff occurs before the first response has peaked. Time bin is 25 ms. (D) Histogram and cumulative probability plot of processing times based on the time to half-maximal activation of the average response across all responding glomeruli in each trial (median, 40 ms). Time bin in 50 ms. In (B), (C), and (D), the end of the interval measured was the time of the second inhalation, as indicated by the gray arrows in the inset.

To more accurately estimate central processing times underlying odor discriminations, we compared presynaptic calcium signal dynamics and behavioral response times on a trial-by-trial basis, using the ISI_1_ value for novel-odorant trials only as the response time measure. For these trials, the time from the onset of the earliest-responding glomerulus (i.e., *t*_detect_) to the beginning of the next inhalation had a mean of 109 (±44) ms (*n* = 21). Thus, ORN input first arrives at the OB 50–150 ms before the discriminative behavioral response begins.

This analysis yields an estimate of the maximum central processing time available after detection of an odorant by ORNs, but is based on response timing in only one glomerulus per trial. However, most models of odor coding rely on patterns of activity across many activated glomeruli [3]. Because these patterns develop over time with each inhalation [6], we next measured available central processing time as a function of different parameters of the overall pattern of ORN inputs to glomeruli sampled across the dorsal OB. We focused on three different parameters: response onset (*t*_onset_, the onset time of the presynaptic calcium signal), peak time (defined as *t*_90_, the time to 90% of the maximum amplitude of the calcium signal), and time to half-maximal activation (*t*_50,avg_) of the aggregate response averaged across all glomeruli. The first two parameters were measured separately for every responding glomerulus, and the last was measured from the averaged response of all activated glomeruli in a trial (see Materials and Methods). The time from each response parameter to the beginning of the next inhalation in novel-odorant trials yielded a distribution of times available for the central processing of odor information.

The distribution of central processing times relative to response onset (*t*_onset_), near-maximal response (*t*_90_), and half-maximal activation of the aggregate signal (*t*_50,avg_) are shown in Figure 5. Processing times relative to response onset were the longest, with a median of 75 (±48) ms (*n* = 196 glomeruli) (Figure 5B). In contrast, processing times relative to near-maximal signal amplitude had a median of only 6 (±63) ms, indicating that many glomeruli reach peak response amplitude *after* the behavioral response has already begun (Figure 5C). The *t*_50,avg_ measure yielded intermediate processing times, with a median of 40 (±90) ms (Figure 5D). These distributions allow for estimates of the information available for odor discrimination as central processing time varies (Figure 6A). For each parameter, the fraction of ORN inputs contributing to odor discrimination drops sharply as processing time increases beyond several tens of milliseconds.

**Figure 6:**
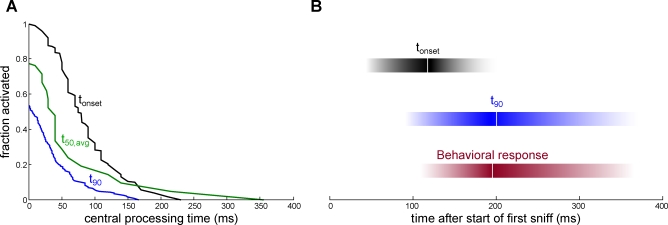
Dynamics of Glomerular Activation Relative to Odorant Response Times (A) Plot of the fraction of glomeruli or trials available to contribute to odor coding as a function of central processing time. Three response parameters are plotted: response onset (*t*_onset_), near-maximal activation (*t*_90_), and half-maximal activation of the aggregate response for one trial (*t*_50_,_avg_). Each plot is derived from the cumulative probability distribution of its processing time (see Figure 5), truncating values that occur after the behavioral response has begun (which would give a negative central processing time). The *t*_onset_ and *t*_90_ plots are pooled across all glomeruli, whereas the *t*_50,avg_ plot is on a per trial basis. See Results for further explanation. (B) Schematic summarizing the dynamics of neural activity and behavioral responses after the first inhalation of a novel odorant: *t*_onset_, time to the earliest arrival of receptor input at the olfactory bulb; *t*_90_, time to 90% signal amplitude for the optical signal in all glomeruli; behavioral response, time to the next inhalation for novel-odorant trials. Solid bars are representations of the probability of occurrence for each parameter. Darker color indicates increased probability; these probability density functions were estimated by fitting Gaussian distributions to the data in Figures 2C, 5A, and 5C. The median is indicated by the white line in the center of each bar.

Errors in this analysis could arise from the approximately one in four trials (see above) in which the rat showed a short ISI_1_ spontaneously rather than in response to the novel odorant. The most conservative correction for this possibility is to eliminate the 25% of trials (5/21) with the shortest ISI_1_ values. Omission of these trials yielded median processing times of 70 ms (*t*_onset_; *n* = 128 glomeruli), 15 ms (*t*_90_; *n* = 128), and 40 ms (*t*_50,avg_; *n* = 16 trials).

## Discussion

### Rapid Spontaneous Discrimination of Novel Odors

Much recent attention has focused on the speed with which animals can perform simple odor discrimination tasks [11–13]. Such information is useful in defining the limits of perceptual performance and in constraining models of the underlying neural mechanisms. In this study, using a unique behavioral readout of odor perception that relies on the animal's natural, unreinforced behavioral response to an odorant, we measured odor discrimination times that were as rapid as those reported in paradigms using highly trained operant responses. Our data also confirm—as shown by others [12–38]—that rats can (and typically do) identify an odorant as novel after a single inhalation: respiratory behavior diverged dramatically for novel- and learned-odorant trials after the first inhalation, with only a single novel trial eliciting a first ISI larger then the median value of all learned-odorant trials. Thus, even without prior training, rats discriminate a novel odorant from a learned one rapidly (<200 ms) and after one sample of the odorant. Similarly rapid odorant-dependent changes in respiration have also been reported in humans, where changes in inhalation strength as a function of odorant intensity emerge in as little as 160 ms for mixed olfactory and trigeminal stimuli, and 260 ms for pure olfactory odorants [30].

Our head-fixed behavioral paradigm differed in several important ways from recent studies measuring odor discrimination times in freely moving rats and mice. First, we measured response times relative to the time of first inhalation of odorant, not the time of odorant onset. Response times relative to odorant onset were considerably longer and more variable (unpublished data) due to the low baseline respiration rate (1–2 Hz) in our paradigm. We confirmed that ORNs do not detect odorant without an inhalation. Prior studies in freely moving rodents have measured response times relative to the time of odorant presentation, and have obtained values ranging from 200–500 ms [11–14,39]. In these paradigms, however, rats begin sniffing at elevated rates of 6–9 Hz just prior to odorant sampling [23,29]; we have observed similar behavior in freely-moving mice (D. Wesson, unpublished data). Assuming a 50% duty cycle of inhalation and exhalation at 8-Hz sniffing (inhalation duration of ∼60 ms [29]), the delay between odorant onset and inhalation would be, on average, approximately 63 ms. This delay may account for the slightly longer discrimination times reported in earlier studies.

Our paradigm also differed in its behavioral readout, relying on an ethologically “natural” response to a novel stimulus [25–28] rather than a shaped behavior. Indeed, response times measured using our operant readout of discrimination of two learned odorants (licking) were considerably longer (median, 700 ms) than either the respiratory response to a novel odorant measured in the same animals and sessions or the response times measured using withdrawal from an odor port in earlier studies [11–14,39]. As is well established, the choice of behavioral response can strongly affect response times, as can other experimental parameters such as task difficulty and stimulus valence [11,16], as well as (presumably) whether an animal is freely moving or restrained. Thus, absolute response times do not solely reflect the time required for processing sensory information; however, *minimal* response times obtained in a given paradigm do effectively set limits on this time period. In this context, it is notable that a variety of different behavioral paradigms all indicate that rodents can perform elementary odor discriminations in approximately 200 ms or less [11–13].

The spontaneous respiration-based assay used in this study also has limitations in probing strategies underlying odor discrimination. For example, such an assay is unlikely to yield insights into the relationship between discrimination speed and accuracy, because it relies on a spontaneous and unrewarded recognition of odorant novelty; thus, the concept of “accuracy” is not clearly defined. This paradigm is also poorly suited to analyzing neural activity or behavioral responses over many (i.e., hundreds or thousands) trials, because only a limited number of novel odorants may be screened per session. Finally, the cognitive rules underlying the perception of an odorant as novel are unclear. Despite these limitations, an odorant-evoked increase in respiration rate reports the perceptual discrimination of one odorant from another with reliability comparable to that of operant paradigms.

It is also possible that the novelty response requires little central processing, whereas discriminating two learned odorants is more demanding and would require more time. For example, rats require more trials to learn to discriminate between aliphatic odorants differing by one carbon in chain length in a two-choice operant task [32,33], and show spontaneous generalization and cross-habituation to such odorant pairs as well as to the some enantiomers [33,34]. In our paradigm, however, rats rapidly responded to the novel member of these same odorant pairs and did so with similar speed and accuracy as for dissimilar novel odorants. It seems unlikely that the initial coding and sensory processing demands should differ in these paradigms, as novelty detection still requires that the novel odorant be discriminated from the learned one. Instead, longer response times or slower learning may reflect differences in the higher-order pathways that underlie different behavioral responses such as inhalation versus licking or head movement. Finally, the fact that rats spontaneously discriminated odorant pairs that they fail to discriminate in freely moving, spontaneous discrimination assays reiterates the fact that discrimination “difficulty”—as well as response time—is highly dependent on the assay used [34].

### Rapid Encoding of Odorant Identity

A key feature of this study is the integration of behavioral response time measurements with simultaneous measurements of the underlying neural activity. These data are summarized in Figure 6B. We provide, for the first time, estimates of the time required for the peripheral events underlying odor detection in the behaving animal (see Figure 5). We found that olfactory information arrives at the brain over a range of 80–160 ms after the start of an inhalation. Given an estimated conduction time of 2–12 ms (based on a conduction velocity of 0.5 m/s [40] and a distance of 1–6 mm), this value suggests that odorant transduction can occur in as little as 70 ms; this estimate is much faster than the 150–600 ms reported for transduction times in ORNs in vitro [41,42]. Transduction might occur more quickly during natural respiration in vivo, although we are not aware of any measurements of mammalian ORN transduction times under such circumstances. Our measurements are consistent, however, with the emergence of odorant-specific response patterns in piriform cortex neurons as soon as 100 ms after inhalation [43,44].

Thus, the bulk of the time required for odor discrimination is due to the process of odorant detection (including transduction and propagation of electrical signals to the OB) by the primary sensory neurons. In fact, the shortest response times we could reliably detect with our paradigm (140 ms) were nearly the same as the average detection time based on the optical signal measurements (∼120 ms). By making a trial-by-trial comparison of detection time and time to next inhalation for novel odor trials, we estimate that the entire central component of the response to a novel odorant—from processing in the OB and downstream targets to motor changes in respiration—requires, conservatively, 75 ± 50 ms. Given that the motor component underlying respiratory changes requires 20–40 ms at minimum [45], these results suggest that extremely little processing time is required to discriminate one odorant from another.

These estimates are based on data obtained from glomeruli of the dorsal OB, which represent approximately 10% of the entire glomerular population. The majority of the novel odorants used (see Materials and Methods) elicit peak or near-peak levels of activity in the dorsal OB in 2-deoxyglucose or functional magnetic resonance imaging (fMRI) mapping studies [46,47]. Others (eugenol and butyl acetate, for example) show no clear 2-deoxyglucose foci on the dorsal OB but have been shown by other methods to potently activate isolated dorsal glomeruli [48,49]. Nonetheless, 2-deoxyglucose and fMRI mapping suggests that all odorants used here almost certainly evoked strong input to non-dorsal glomeruli [46,47]. However it seems unlikely that our estimates of odorant detection times would shorten substantially had non-dorsal glomeruli been included in the analysis. Differences in axonal projection distance of as high as 8 mm (a maximal estimate of the distance from the anteroventral to posterodorsal OB [50]) would result in conduction time differences of, at most, approximately 16 ms. Furthermore, detection times measured from the dorsal OB are already below the limit expected from reported odorant transduction delays [41,42]. Nonetheless, our conclusions from dorsal OB glomeruli require further testing in order to characterize odor perception times relative to the dynamics of neural activity in other OB locations, which are potentially more strongly activated. Such tests could be done using chronic electrode recordings in behaving animals, for example.

### Strategies for Rapid Coding and Processing of Odor Information

What do these results imply about strategies for encoding and processing olfactory information? To constrain potential models for odor coding, we estimated the time available for odor discrimination relative to the dynamics of ORN input to the OB using different parameters of the imaged response. Our choice of measured parameters was roughly guided by models of how odor information might be encoded at the level of OB glomeruli. One common model postulates that odor identity is encoded in the relative magnitudes of activation across many glomeruli [2]; characterizations of these relative patterns—or maps—typically focus on peak activation levels, expressed in terms of action potential firing rate or optical or metabolic signal size [3]. To test this model, we measured processing time relative to the time at which ORN inputs reach 90% of their maximal amplitude. Surprisingly, over 40% of all glomeruli reached peak activation *after* the behavioral response to the odorant had already begun. Thus, in most trials, the rat has already identified an odorant as novel and begun responding to it before peak activity maps have fully developed. If as little as 50 ms is required for all subsequent central events—including firing of respiratory motoneurons—less than 20% of all glomeruli have reached peak activation within the requisite time-frame. Thus, comparing peak activity levels across glomeruli is an unlikely strategy for performing elementary odor discriminations.

A variant of this model relies on the most strongly activated glomeruli to encode odor identity [2]. Because different glomeruli are activated with different latencies [6], such a model requires that the aggregate activity of all responsive glomeruli reach high levels in order for strongly activated glomeruli to differentiate from weakly activated ones. We chose the time to half-maximal activation of the aggregate ORN response across all glomeruli signal to estimate this time. This parameter yielded longer central processing times; nonetheless, aggregate activity had reached half-maximal levels in under 40% of all trials. “Reading” activity maps at lower aggregate response levels would yield longer processing times, but at the expense of a reduction in signal-to-noise ratio.

Other models of odor coding postulate that the temporal dynamics of glomerular activation encode odor identity [4,5,51]. To constrain these models, we measured available processing time relative to the time of onset of the ORN signal in responsive glomeruli. This parameter yielded the longest processing times, with approximately 80% of glomeruli activated within a time window allowing 50 ms for the central component of odor discrimination.

This analysis constrains potential models of odor coding as well as the interpretation of experimental data. For example, earlier studies of odor discrimination times [11–13] have noted that a short response time limits odor coding schemes that rely on the temporal dynamics of neural activity over several hundred milliseconds or more [15,52]. However, our results also limit the degree to which the well-characterized *spatial* patterns of glomerular activity might participate in odor coding, because these patterns themselves are temporally dynamic and require up to several hundred milliseconds to develop [6]. Nearly all prior characterizations of odor representations by activity maps rely on responses measured at their peak amplitudes [35,53] or on activity integrated over even longer time periods [54]. Likewise, peak firing rates of mitral/tufted cells—the main output neurons of the OB—integrated over a second or more have also been used to characterize odor coding strategies at the postsynaptic level [55,56]. Our results suggests that activity maps focused on peak or time-integrated activity may be unreliable representatives of the patterns of neural activation occurring at the time of odor discrimination. More generally, the extremely short time required for the central processing of odor information sharply limits the role that coding strategies based on changes in firing rate can play in odor identification.

In contrast, our data are compatible with a model in which the relative timing (i.e., sequence) of activation of glomeruli encodes odorant identity [5,51]. Robust encoding of odorant identity in the timing of activity relative to the respiratory or theta cycle would require only a fraction of the time of one inhalation, or several tens of milliseconds [5,57]. In such a model, response onset could be encoded by postsynaptic neurons as a change in spike timing or synchrony [5]. For these spike-timing–based strategies, the high spontaneous firing rates observed in mitral/tufted cells [56,58,59] may actually increase the speed with which odorant-specific ensemble codes can be established. The integrative properties of neurons in piriform cortex appear to be well suited to detecting synchronous inputs from small ensembles of OB afferents [60]. Another model consistent with our data is that the earliest-activated glomeruli preferentially contribute to odor identity coding, either in a combinatorial fashion or through a rank order mechanism. Similar strategies likely underlie rapid processing in the somatosensory and visual systems, in which perception of complex stimuli occurs too rapidly to support rate coding as a reliable mechanism [61,62]. In this context, it is interesting to note that the earliest-activated glomeruli are not necessarily those that are the most strongly activated in time-averaged activity maps [6,7].

Regardless of mechanism, the speed with which rats can recognize and respond to a novel odorant, even without prior reinforcement to do so, suggests that the perception of odor identity may be one of the least demanding aspects of olfaction. This may especially be the case in identifying “simple” odors consisting of one or a few components presented in a more or less binary fashion. Such elementary discriminations in other sensory modalities occur with comparable speed, whereas more complex tasks take longer and benefit from integrating sensory information over time [16,17]. Thus, different coding strategies—including any or all of those listed above—may be used depending on the nature of the task itself. Finally, most natural odor-based decisions are likely to involve integrating input across multiple samples (sniffs) as the animal moves through the heterogeneous and dynamic olfactory landscape that surrounds it. For these types of tasks, slower temporal patterning of activity [15,52,63], changes in odor representations as a function of sniffing behavior [22], and rapid context-dependent modulation of neuronal responsiveness [58] may play important roles.

## Materials and Methods

Data were acquired from 13 adult female Long-Evans rats. A different dataset from a subset of these animals has been published in an earlier study [22]; all surgical, behavioral, and recording procedures are as described in that study. For clarity, these are outlined again in brief below.

### Head-fixed behavioral paradigm.

Naive animals were outfitted with a head bolt for restraint [21] and an intranasal cannula for chronic measurement of respiration in a single surgical procedure. Behavioral training began 1–2 wk after surgery. Rats were water-deprived and habituated to restraint by providing intermittent water reward through a lick spout. Rats were then shaped to discriminate a rewarded odorant (CS+) from an unrewarded odorant (CS−) by licking the lick spout in response to the CS+ and refraining from licking to the CS−. In the final discrimination paradigm, odorants were presented for 4–5.5 s with an intertrial interval (ITI) varying randomly from 15–24 s. A tone preceded odorant presentation by 1 s. Incorrect licking at any time during presentation of the CS− was punished with a 7-s increase in the ITI. After the full training sequence, rats were run in a single daily session consisting of 50–140 trials (30–60 min). Behavioral data from “standard” CS− trials were used to represent baseline sniffing behavior in this study. Except for the report of lick times, data from CS+ trials were not included in any analyses.

Occasionally in a session, the standard CS− odorant was replaced with an odorant not previously experienced by the rat in the last 48 h (a *novel* odorant). This odorant was then presented two to six additional times, either consecutively or interspersed with CS+ presentations. Not more than four novel odorants (typically one to two) were presented in a single session. Licking in response to a novel odorant occurred in approximately 20% of trials; rats were not rewarded for licking to the novel odorant.

### Optical recordings.

ORN input to the dorsal OB of head-fixed rats was imaged by loading ORNs with calcium-sensitive dye as described previously [22–35]. After behavioral training and 1 d prior to imaging, an optical window was placed over the dorsal surface of both OBs by thinning the overlying bone and sealing with ethyl-2-cyanoacrylate glue. Optical signals were recorded in select trials during the next one to five behavioral sessions. Signals were collected using an Olympus epifluorescence illumination turret (BX51) and full light from a 150W Xenon arc lamp (Opti-Quip) and appropriate filter sets [22]. The imaged area covered a region of approximately 3 mm (anterior-posterior) × 1.5 mm (medial-lateral) over one OB. Images were acquired using a 256 × 256 pixel charge-coupled device (CCD) camera and digitized at 25 Hz along with respiration and behavioral (licking) signals using an integrated hardware/software package (NeuroCCD SM-256 and NeuroPlex; RedShirtImaging).

### Olfactometry.

Odorants were monomolecular hydrocarbon compounds known to reliably evoke ORN input to dorsal OB glomeruli [35,54]. Nonimaged novel and learned odorants are specified in Table 1. Imaged novel odorants, along with the number of novel presentations of each, were benzaldehyde (*n =* 2), 2-butanone (*n =*2), butyl acetate (*n =*1), butyraldehyde (*n =*1), ethyl butyrate (*n =*7), eugenol (*n =*3), 2-hexanone (*n =*2), methyl benzoate (*n =*1), and valeric acid (*n =*2). All odorants were presented using a custom, computer-controlled flow-dilution olfactometer that allowed precise control of odorant concentration, identity, and onset timing [22]. Concentrations are reported as percent dilution of saturated vapor (s.v.), and ranged from 0.5%–5% (1% s.v. was typical).

### Data analysis.

Respiration was measured by connecting the intranasal cannula to a pressure sensor (Honeywell, model# 24PCAFA6G) with PE tubing. Pressure decreases appeared as positive voltages. Intranasal pressure was amplified 100×, high-pass filtered at 1 kHz, then digitized at 100–500 Hz in synchrony with optical signals. To identify the start time of each inhalation, signals were band-pass filtered (second-order Butterworth; 1–25 Hz) and integrated. Each valley in this waveform signaled the start of a decrease in intranasal pressure; if this signal increased past an empirically derived threshold within 30 ms of the valley, the valley was identified as an inhalation. In simultaneous recordings of both intranasal pressure and airflow (measured with a thermocouple, see [22]), we found that this time corresponded very well with the time of the onset of airflow into the dorsal recess.

Initial processing of optical signals was performed as described previously [22]. Briefly, optical signals were first processed to remove widespread intrinsic signals and movement artifacts, then regions of interest (ROIs) representing one or a few glomeruli were chosen and signals spatially averaged across each ROI. ROIs were chosen from “sniff-triggered average” response maps by visual inspection, and were chosen to be, on average, slightly smaller than the half-width of the underlying optical signal focus. Signals digitized at 25 Hz were up-sampled to 100 Hz to match the respiration signal for easier analysis. Thus, temporal precision of measurements based on the optical signals was 10 ms, but only included temporal frequencies below 12.5 Hz. Measurements using optical signals sampled directly at 100 Hz (50 Hz Nyquist frequency) yielded equivalent detection times (unpublished data, but see [22]).

A custom algorithm was developed to automatically detect and characterize the dynamics of respiration-evoked optical signals from each ROI. This analysis allowed robust and objective measurement of response timing and amplitudes. First, the signal from each ROI was further denoised by band-pass filtering (second-order Butterworth, 0.4–8 Hz) followed by wavelet decomposition-based denoising using standard Matlab functions (fourth-order Daubechies wavelet decomposition, soft thresholding of the coefficients at level 3, and then reconstruction). The start of a response was identified by a strong upwards inflection in the optical signal waveform, defined as the first peak after each sniff in the product of the filtered signal's first and second derivatives (i.e., the time at which the slope and concavity were the greatest). Starting at this time, each response was fitted (least-squares curve fitting) with a double-sigmoid function (a sigmoid rise followed by a sigmoid fall). The time (and amplitude) of the peak of this response was defined as the peak in this fitted response function, rather than the peak of the raw optical signal. The signal-to-noise ratio of the response was defined as the fitted peak amplitude divided by the “noise”. The noise for each ROI was defined as the standard deviation of the amplitudes of the responses evoked by all inhalations during periods in between odorant presentations. This measure was used to identify strongly responsive ROIs. Detection and processing times for a trial were based on the response times for each ROI, pooled across trials. The number of ROIs contributing to the novel-odorant analysis (21 trials) for each of the seven rats was: 22, 38, 10, 25, 54, 24, 22.

All analysis was performed using custom software written in Matlab or LabView. Statistical tests were performed with Matlab. All data distributions were tested for normality (Kolmogorov-Smirnov test) before reporting mean or median values and choosing appropriate statistical tests.

## Abbreviations

CS+: rewarded odorant
CS−: unrewarded odorant
ISI: intersniff interval
OB: olfactory bulb
ON: olfactory nerve
ORN: olfactory receptor neuron
ROC: receiver operating characteristic
ROI: region of interest
